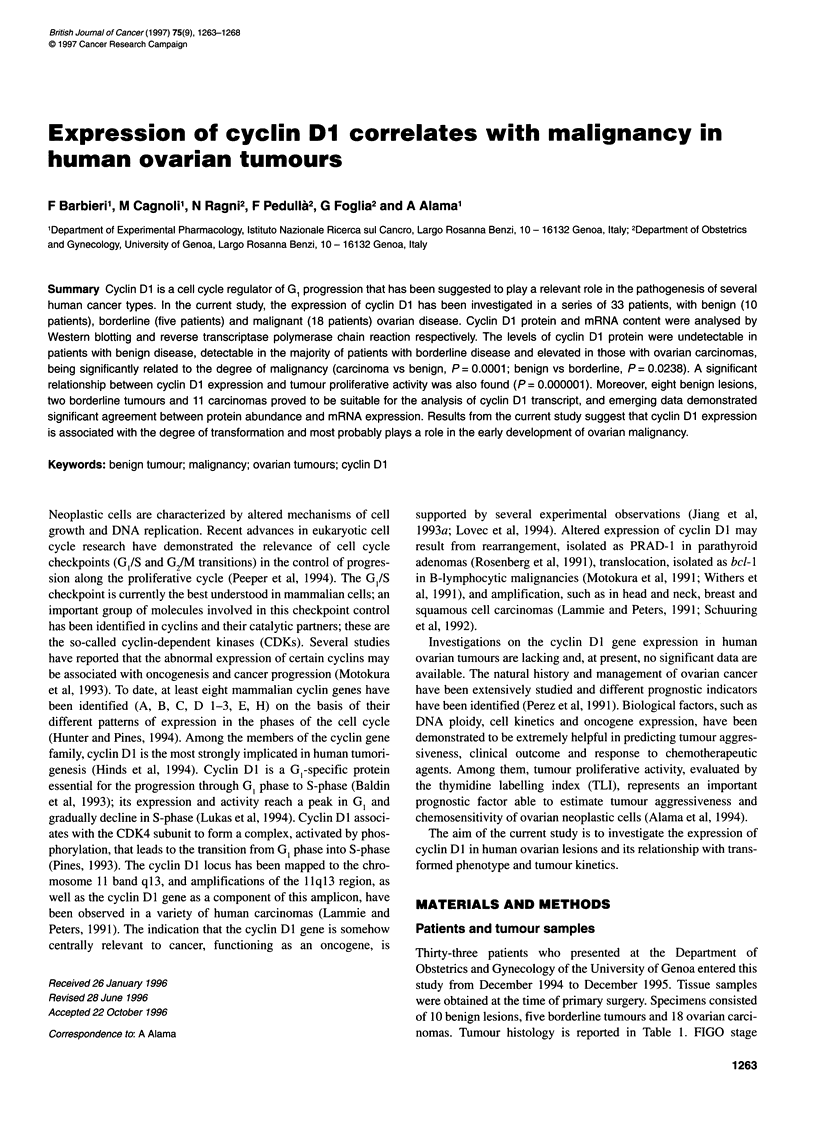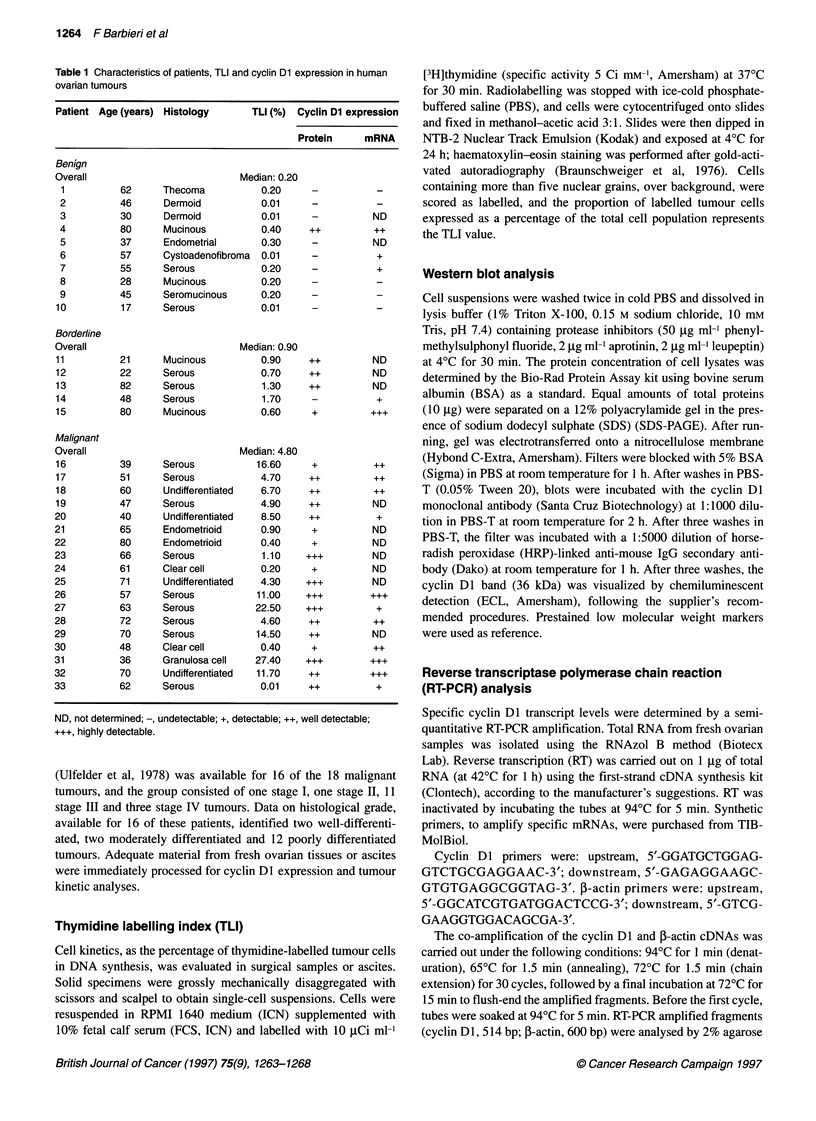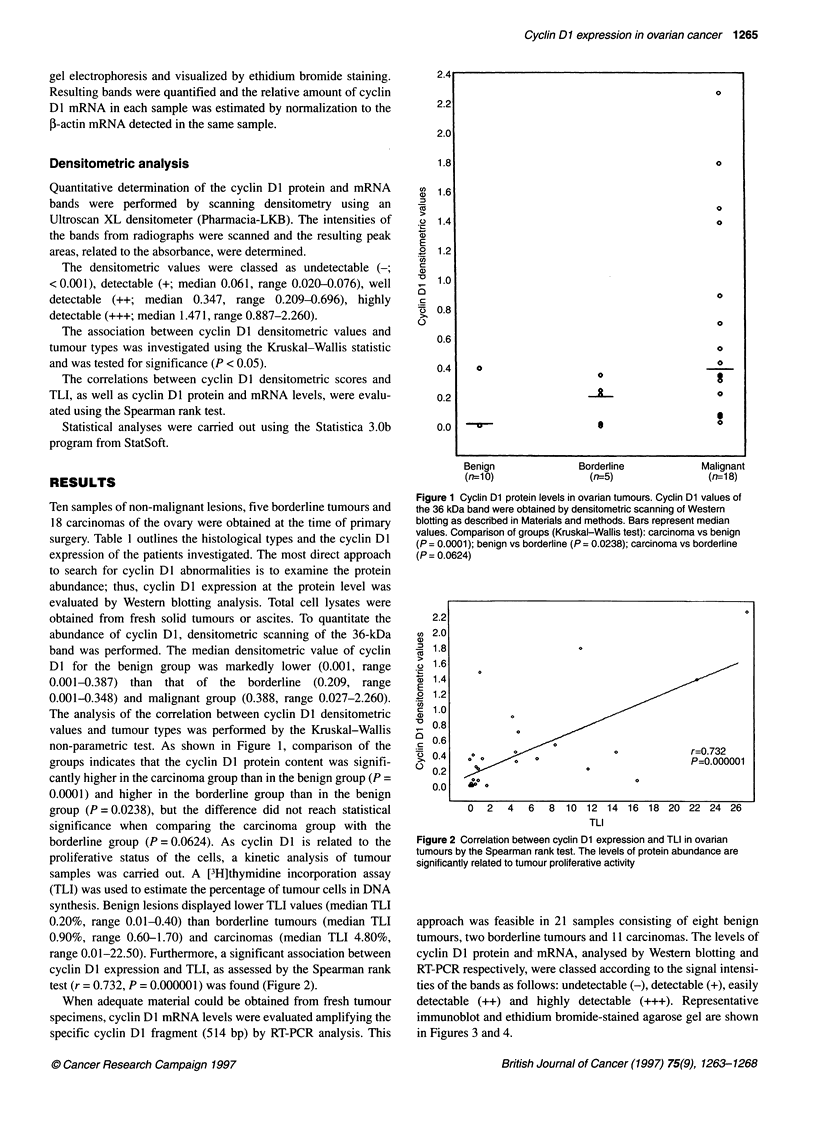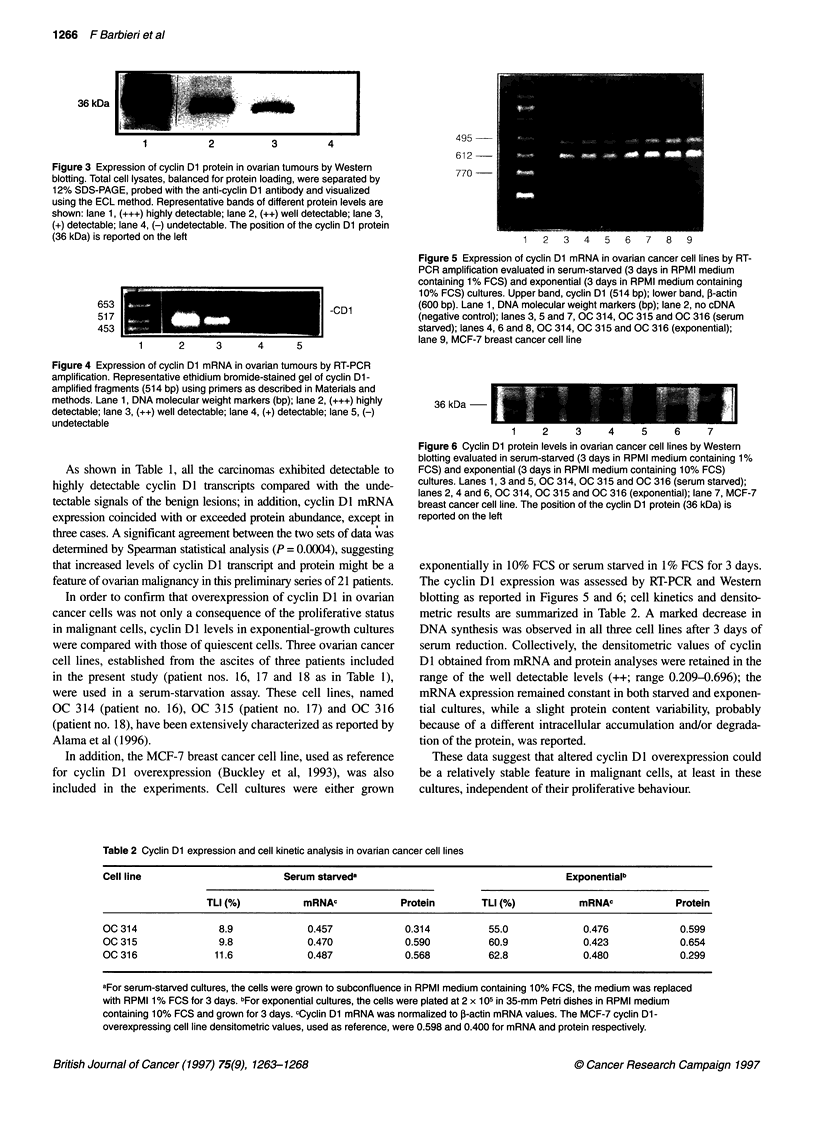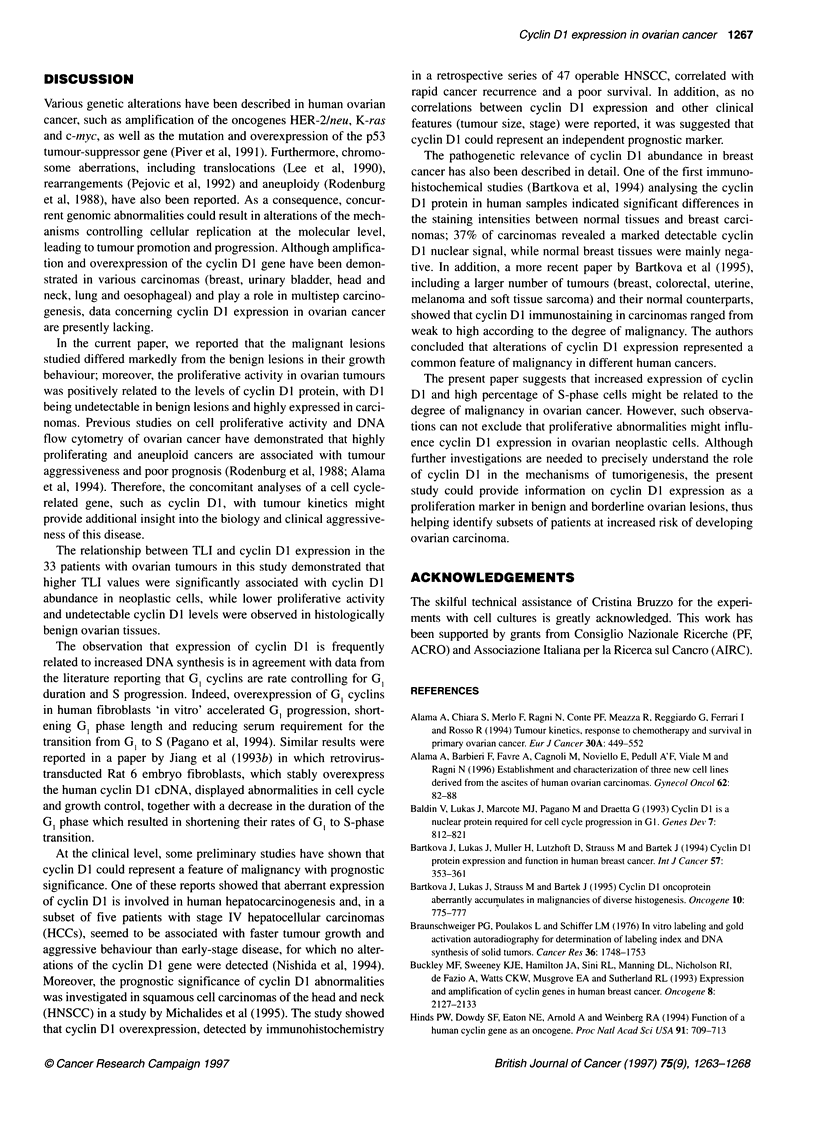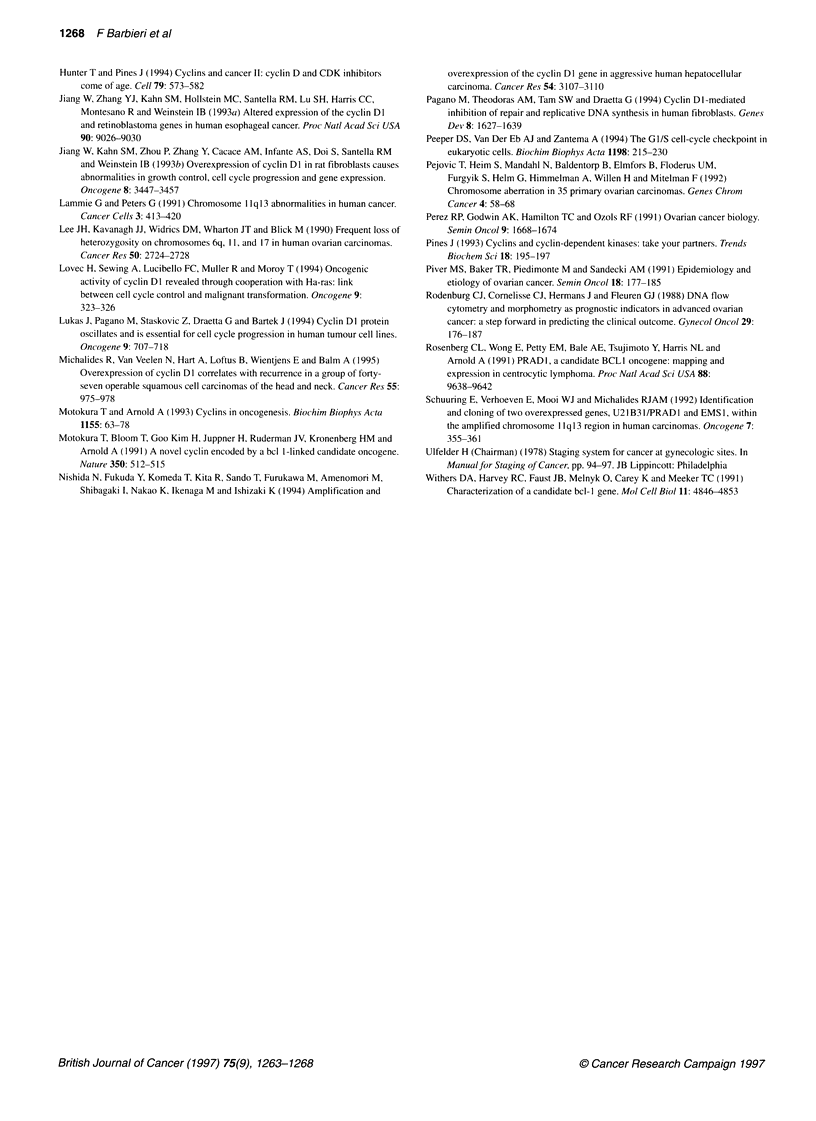# Expression of cyclin D1 correlates with malignancy in human ovarian tumours.

**DOI:** 10.1038/bjc.1997.215

**Published:** 1997

**Authors:** F. Barbieri, M. Cagnoli, N. Ragni, F. PedullÃ , G. Foglia, A. Alama

**Affiliations:** Department of Experimental Pharmacology, Istituto Nazionale Ricerca sul Cancro, Largo Rosanna Benzi, Genoa, Italy.

## Abstract

**Images:**


British Journal of Cancer (1997) 75(9), 1263–1268
© 1997 Cancer Research Campaign

### Expression of cyclin D1 correlates with malignancy in human ovarian tumours

F Barbieri[1], M Cagnoli[1], N Ragni[2], F Pedullà[2], G Foglia[2] and A Alama[1]

[1]Department of Experimental Pharmacology, Istituto Nazionale Ricerca sul Cancro, Largo Rosanna Benzi, 10 – 16132 Genoa, Italy; [2]Department of Obstetrics and Gynecology, University of Genoa, Largo Rosanna Benzi, 10 – 16132 Genoa, Italy

**Summary** Cyclin D1 is a cell cycle regulator of $G_1$ progression that has been suggested to play a relevant role in the pathogenesis of several human cancer types. In the current study, the expression of cyclin D1 has been investigated in a series of 33 patients, with benign (10 patients), borderline (five patients) and malignant (18 patients) ovarian disease. Cyclin D1 protein and mRNA content were analysed by Western blotting and reverse transcriptase polymerase chain reaction respectively. The levels of cyclin D1 protein were undetectable in patients with benign disease, detectable in the majority of patients with borderline disease and elevated in those with ovarian carcinomas, being significantly related to the degree of malignancy (carcinoma vs benign, $P = 0.0001$; benign vs borderline, $P = 0.0238$). A significant relationship between cyclin D1 expression and tumour proliferative activity was also found ($P = 0.000001$). Moreover, eight benign lesions, two borderline tumours and 11 carcinomas proved to be suitable for the analysis of cyclin D1 transcript, and emerging data demonstrated significant agreement between protein abundance and mRNA expression. Results from the current study suggest that cyclin D1 expression is associated with the degree of transformation and most probably plays a role in the early development of ovarian malignancy.

**Keywords:** benign tumour; malignancy; ovarian tumours; cyclin D1

Neoplastic cells are characterized by altered mechanisms of cell growth and DNA replication. Recent advances in eukaryotic cell cycle research have demonstrated the relevance of cell cycle checkpoints ($G_1$/S and $G_2$/M transitions) in the control of progression along the proliferative cycle (Peeper et al, 1994). The $G_1$/S checkpoint is currently the best understood in mammalian cells; an important group of molecules involved in this checkpoint control has been identified in cyclins and their catalytic partners; these are the so-called cyclin-dependent kinases (CDKs). Several studies have reported that the abnormal expression of certain cyclins may be associated with oncogenesis and cancer progression (Motokura et al, 1993). To date, at least eight mammalian cyclin genes have been identified (A, B, C, D 1–3, E, H) on the basis of their different patterns of expression in the phases of the cell cycle (Hunter and Pines, 1994). Among the members of the cyclin gene family, cyclin D1 is the most strongly implicated in human tumorigenesis (Hinds et al, 1994). Cyclin D1 is a $G_1$-specific protein essential for the progression through $G_1$ phase to S-phase (Baldin et al, 1993); its expression and activity reach a peak in $G_1$ and gradually decline in S-phase (Lukas et al, 1994). Cyclin D1 associates with the CDK4 subunit to form a complex, activated by phosphorylation, that leads to the transition from $G_1$ phase into S-phase (Pines, 1993). The cyclin D1 locus has been mapped to the chromosome 11 band q13, and amplifications of the 11q13 region, as well as the cyclin D1 gene as a component of this amplicon, have been observed in a variety of human carcinomas (Lammie and Peters, 1991). The indication that the cyclin D1 gene is somehow centrally relevant to cancer, functioning as an oncogene, is supported by several experimental observations (Jiang et al, 1993*a*; Lovec et al, 1994). Altered expression of cyclin D1 may result from rearrangement, isolated as PRAD-1 in parathyroid adenomas (Rosenberg et al, 1991), translocation, isolated as *bcl*-1 in B-lymphocytic malignancies (Motokura et al, 1991; Withers et al, 1991), and amplification, such as in head and neck, breast and squamous cell carcinomas (Lammie and Peters, 1991; Schuuring et al, 1992).

Investigations on the cyclin D1 gene expression in human ovarian tumours are lacking and, at present, no significant data are available. The natural history and management of ovarian cancer have been extensively studied and different prognostic indicators have been identified (Perez et al, 1991). Biological factors, such as DNA ploidy, cell kinetics and oncogene expression, have been demonstrated to be extremely helpful in predicting tumour aggressiveness, clinical outcome and response to chemotherapeutic agents. Among them, tumour proliferative activity, evaluated by the thymidine labelling index (TLI), represents an important prognostic factor able to estimate tumour aggressiveness and chemosensitivity of ovarian neoplastic cells (Alama et al, 1994).

The aim of the current study is to investigate the expression of cyclin D1 in human ovarian lesions and its relationship with transformed phenotype and tumour kinetics.

### MATERIALS AND METHODS

### Patients and tumour samples

Thirty-three patients who presented at the Department of Obstetrics and Gynecology of the University of Genoa entered this study from December 1994 to December 1995. Tissue samples were obtained at the time of primary surgery. Specimens consisted of 10 benign lesions, five borderline tumours and 18 ovarian carcinomas. Tumour histology is reported in Table 1. FIGO stage

*Received 26 January 1996*
*Revised 28 June 1996*
*Accepted 22 October 1996*

*Correspondence to:* A Alama

**Table 1** Characteristics of patients, TLI and cyclin D1 expression in human ovarian tumours

| Patient | Age (years) | Histology | TLI (%) | Cyclin D1 expression | |
|---|---|---|---|---|---|
| | | | | Protein | mRNA |
| *Benign* | | | | | |
| Overall | | | Median: 0.20 | | |
| 1 | 62 | Thecoma | 0.20 | – | – |
| 2 | 46 | Dermoid | 0.01 | – | – |
| 3 | 30 | Dermoid | 0.01 | – | ND |
| 4 | 80 | Mucinous | 0.40 | ++ | ++ |
| 5 | 37 | Endometrial | 0.30 | – | ND |
| 6 | 57 | Cystoadenofibroma | 0.01 | – | + |
| 7 | 55 | Serous | 0.20 | – | + |
| 8 | 28 | Mucinous | 0.20 | – | – |
| 9 | 45 | Seromucinous | 0.20 | – | – |
| 10 | 17 | Serous | 0.01 | – | – |
| *Borderline* | | | | | |
| Overall | | | Median: 0.90 | | |
| 11 | 21 | Mucinous | 0.90 | ++ | ND |
| 12 | 22 | Serous | 0.70 | ++ | ND |
| 13 | 82 | Serous | 1.30 | ++ | ND |
| 14 | 48 | Serous | 1.70 | – | + |
| 15 | 80 | Mucinous | 0.60 | + | +++ |
| *Malignant* | | | | | |
| Overall | | | Median: 4.80 | | |
| 16 | 39 | Serous | 16.60 | + | ++ |
| 17 | 51 | Serous | 4.70 | ++ | ++ |
| 18 | 60 | Undifferentiated | 6.70 | ++ | ++ |
| 19 | 47 | Serous | 4.90 | ++ | ND |
| 20 | 40 | Undifferentiated | 8.50 | ++ | + |
| 21 | 65 | Endometrioid | 0.90 | + | ND |
| 22 | 80 | Endometrioid | 0.40 | + | ND |
| 23 | 66 | Serous | 1.10 | +++ | ND |
| 24 | 61 | Clear cell | 0.20 | + | ND |
| 25 | 71 | Undifferentiated | 4.30 | +++ | ND |
| 26 | 57 | Serous | 11.00 | +++ | +++ |
| 27 | 63 | Serous | 22.50 | +++ | + |
| 28 | 72 | Serous | 4.60 | ++ | ++ |
| 29 | 70 | Serous | 14.50 | ++ | ND |
| 30 | 48 | Clear cell | 0.40 | + | ++ |
| 31 | 36 | Granulosa cell | 27.40 | +++ | +++ |
| 32 | 70 | Undifferentiated | 11.70 | ++ | +++ |
| 33 | 62 | Serous | 0.01 | ++ | + |

ND, not determined; –, undetectable; +, detectable; ++, well detectable; +++, highly detectable.

(Ulfelder et al, 1978) was available for 16 of the 18 malignant tumours, and the group consisted of one stage I, one stage II, 11 stage III and three stage IV tumours. Data on histological grade, available for 16 of these patients, identified two well-differentiated, two moderately differentiated and 12 poorly differentiated tumours. Adequate material from fresh ovarian tissues or ascites were immediately processed for cyclin D1 expression and tumour kinetic analyses.

### Thymidine labelling index (TLI)

Cell kinetics, as the percentage of thymidine-labelled tumour cells in DNA synthesis, was evaluated in surgical samples or ascites. Solid specimens were grossly mechanically disaggregated with scissors and scalpel to obtain single-cell suspensions. Cells were resuspended in RPMI 1640 medium (ICN) supplemented with 10% fetal calf serum (FCS, ICN) and labelled with 10 μCi $ml^{-1}$ [$^3$H]thymidine (specific activity 5 Ci $mM^{-1}$, Amersham) at 37°C for 30 min. Radiolabelling was stopped with ice-cold phosphate-buffered saline (PBS), and cells were cytocentrifuged onto slides and fixed in methanol–acetic acid 3:1. Slides were then dipped in NTB-2 Nuclear Track Emulsion (Kodak) and exposed at 4°C for 24 h; haematoxylin–eosin staining was performed after gold-activated autoradiography (Braunschweiger et al, 1976). Cells containing more than five nuclear grains, over background, were scored as labelled, and the proportion of labelled tumour cells expressed as a percentage of the total cell population represents the TLI value.

### Western blot analysis

Cell suspensions were washed twice in cold PBS and dissolved in lysis buffer (1% Triton X-100, 0.15 M sodium chloride, 10 mM Tris, pH 7.4) containing protease inhibitors (50 μg $ml^{-1}$ phenylmethylsulphonyl fluoride, 2 μg $ml^{-1}$ aprotinin, 2 μg $ml^{-1}$ leupeptin) at 4°C for 30 min. The protein concentration of cell lysates was determined by the Bio-Rad Protein Assay kit using bovine serum albumin (BSA) as a standard. Equal amounts of total proteins (10 μg) were separated on a 12% polyacrylamide gel in the presence of sodium dodecyl sulphate (SDS) (SDS-PAGE). After running, gel was electrotransferred onto a nitrocellulose membrane (Hybond C-Extra, Amersham). Filters were blocked with 5% BSA (Sigma) in PBS at room temperature for 1 h. After washes in PBS-T (0.05% Tween 20), blots were incubated with the cyclin D1 monoclonal antibody (Santa Cruz Biotechnology) at 1:1000 dilution in PBS-T at room temperature for 2 h. After three washes in PBS-T, the filter was incubated with a 1:5000 dilution of horseradish peroxidase (HRP)-linked anti-mouse IgG secondary antibody (Dako) at room temperature for 1 h. After three washes, the cyclin D1 band (36 kDa) was visualized by chemiluminescent detection (ECL, Amersham), following the supplier's recommended procedures. Prestained low molecular weight markers were used as reference.

### Reverse transcriptase polymerase chain reaction (RT-PCR) analysis

Specific cyclin D1 transcript levels were determined by a semi-quantitative RT-PCR amplification. Total RNA from fresh ovarian samples was isolated using the RNAzol B method (Biotecx Lab). Reverse transcription (RT) was carried out on 1 μg of total RNA (at 42°C for 1 h) using the first-strand cDNA synthesis kit (Clontech), according to the manufacturer's suggestions. RT was inactivated by incubating the tubes at 94°C for 5 min. Synthetic primers, to amplify specific mRNAs, were purchased from TIB-MolBiol.

Cyclin D1 primers were: upstream, 5′-GGATGCTGGAG-GTCTGCGAGGAAC-3′; downstream, 5′-GAGAGGAAGC-GTGTGAGGCGGTAG-3′. β-actin primers were: upstream, 5′-GGCATCGTGATGGACTCCG-3′; downstream, 5′-GTCG-GAAGGTGGACAGCGA-3′.

The co-amplification of the cyclin D1 and β-actin cDNAs was carried out under the following conditions: 94°C for 1 min (denaturation), 65°C for 1.5 min (annealing), 72°C for 1.5 min (chain extension) for 30 cycles, followed by a final incubation at 72°C for 15 min to flush-end the amplified fragments. Before the first cycle, tubes were soaked at 94°C for 5 min. RT-PCR amplified fragments (cyclin D1, 514 bp; β-actin, 600 bp) were analysed by 2% agarose

© Cancer Research Campaign 1997

gel electrophoresis and visualized by ethidium bromide staining. Resulting bands were quantified and the relative amount of cyclin D1 mRNA in each sample was estimated by normalization to the β-actin mRNA detected in the same sample.

### Densitometric analysis

Quantitative determination of the cyclin D1 protein and mRNA bands were performed by scanning densitometry using an Ultroscan XL densitometer (Pharmacia-LKB). The intensities of the bands from radiographs were scanned and the resulting peak areas, related to the absorbance, were determined.

The densitometric values were classed as undetectable (–; < 0.001), detectable (+; median 0.061, range 0.020–0.076), well detectable (++; median 0.347, range 0.209–0.696), highly detectable (+++; median 1.471, range 0.887–2.260).

The association between cyclin D1 densitometric values and tumour types was investigated using the Kruskal–Wallis statistic and was tested for significance ($P < 0.05$).

The correlations between cyclin D1 densitometric scores and TLI, as well as cyclin D1 protein and mRNA levels, were evaluated using the Spearman rank test.

Statistical analyses were carried out using the Statistica 3.0b program from StatSoft.

### RESULTS

Ten samples of non-malignant lesions, five borderline tumours and 18 carcinomas of the ovary were obtained at the time of primary surgery. Table 1 outlines the histological types and the cyclin D1 expression of the patients investigated. The most direct approach to search for cyclin D1 abnormalities is to examine the protein abundance; thus, cyclin D1 expression at the protein level was evaluated by Western blotting analysis. Total cell lysates were obtained from fresh solid tumours or ascites. To quantitate the abundance of cyclin D1, densitometric scanning of the 36-kDa band was performed. The median densitometric value of cyclin D1 for the benign group was markedly lower (0.001, range 0.001–0.387) than that of the borderline (0.209, range 0.001–0.348) and malignant group (0.388, range 0.027–2.260). The analysis of the correlation between cyclin D1 densitometric values and tumour types was performed by the Kruskal–Wallis non-parametric test. As shown in Figure 1, comparison of the groups indicates that the cyclin D1 protein content was significantly higher in the carcinoma group than in the benign group ($P$ = 0.0001) and higher in the borderline group than in the benign group ($P = 0.0238$), but the difference did not reach statistical significance when comparing the carcinoma group with the borderline group ($P = 0.0624$). As cyclin D1 is related to the proliferative status of the cells, a kinetic analysis of tumour samples was carried out. A [$^3$H]thymidine incorporation assay (TLI) was used to estimate the percentage of tumour cells in DNA synthesis. Benign lesions displayed lower TLI values (median TLI 0.20%, range 0.01–0.40) than borderline tumours (median TLI 0.90%, range 0.60–1.70) and carcinomas (median TLI 4.80%, range 0.01–22.50). Furthermore, a significant association between cyclin D1 expression and TLI, as assessed by the Spearman rank test ($r = 0.732$, $P = 0.000001$) was found (Figure 2).

When adequate material could be obtained from fresh tumour specimens, cyclin D1 mRNA levels were evaluated amplifying the specific cyclin D1 fragment (514 bp) by RT-PCR analysis. This approach was feasible in 21 samples consisting of eight benign tumours, two borderline tumours and 11 carcinomas. The levels of cyclin D1 protein and mRNA, analysed by Western blotting and RT-PCR respectively, were classed according to the signal intensities of the bands as follows: undetectable (–), detectable (+), easily detectable (++) and highly detectable (+++). Representative immunoblot and ethidium bromide-stained agarose gel are shown in Figures 3 and 4.

**Figure 1** Cyclin D1 protein levels in ovarian tumours. Cyclin D1 values of the 36 kDa band were obtained by densitometric scanning of Western blotting as described in Materials and methods. Bars represent median values. Comparison of groups (Kruskal–Wallis test): carcinoma vs benign ($P$ = 0.0001); benign vs borderline ($P$ = 0.0238); carcinoma vs borderline ($P$ = 0.0624)

**Figure 2** Correlation between cyclin D1 expression and TLI in ovarian tumours by the Spearman rank test. The levels of protein abundance are significantly related to tumour proliferative activity

© Cancer Research Campaign 1997 

**Figure 3** Expression of cyclin D1 protein in ovarian tumours by Western blotting. Total cell lysates, balanced for protein loading, were separated by 12% SDS-PAGE, probed with the anti-cyclin D1 antibody and visualized using the ECL method. Representative bands of different protein levels are shown: lane 1, (+++) highly detectable; lane 2, (++) well detectable; lane 3, (+) detectable; lane 4, (–) undetectable. The position of the cyclin D1 protein (36 kDa) is reported on the left

**Figure 4** Expression of cyclin D1 mRNA in ovarian tumours by RT-PCR amplification. Representative ethidium bromide-stained gel of cyclin D1-amplified fragments (514 bp) using primers as described in Materials and methods. Lane 1, DNA molecular weight markers (bp); lane 2, (+++) highly detectable; lane 3, (++) well detectable; lane 4, (+) detectable; lane 5, (–) undetectable

As shown in Table 1, all the carcinomas exhibited detectable to highly detectable cyclin D1 transcripts compared with the undetectable signals of the benign lesions; in addition, cyclin D1 mRNA expression coincided with or exceeded protein abundance, except in three cases. A significant agreement between the two sets of data was determined by Spearman statistical analysis ($P = 0.0004$), suggesting that increased levels of cyclin D1 transcript and protein might be a feature of ovarian malignancy in this preliminary series of 21 patients.

In order to confirm that overexpression of cyclin D1 in ovarian cancer cells was not only a consequence of the proliferative status in malignant cells, cyclin D1 levels in exponential-growth cultures were compared with those of quiescent cells. Three ovarian cancer cell lines, established from the ascites of three patients included in the present study (patient nos. 16, 17 and 18 as in Table 1), were used in a serum-starvation assay. These cell lines, named OC 314 (patient no. 16), OC 315 (patient no. 17) and OC 316 (patient no. 18), have been extensively characterized as reported by Alama et al (1996).

In addition, the MCF-7 breast cancer cell line, used as reference for cyclin D1 overexpression (Buckley et al, 1993), was also included in the experiments. Cell cultures were either grown exponentially in 10% FCS or serum starved in 1% FCS for 3 days. The cyclin D1 expression was assessed by RT-PCR and Western blotting as reported in Figures 5 and 6; cell kinetics and densitometric results are summarized in Table 2. A marked decrease in DNA synthesis was observed in all three cell lines after 3 days of serum reduction. Collectively, the densitometric values of cyclin D1 obtained from mRNA and protein analyses were retained in the range of the well detectable levels (++; range 0.209–0.696); the mRNA expression remained constant in both starved and exponential cultures, while a slight protein content variability, probably because of a different intracellular accumulation and/or degradation of the protein, was reported.

These data suggest that altered cyclin D1 overexpression could be a relatively stable feature in malignant cells, at least in these cultures, independent of their proliferative behaviour.

**Figure 5** Expression of cyclin D1 mRNA in ovarian cancer cell lines by RT-PCR amplification evaluated in serum-starved (3 days in RPMI medium containing 1% FCS) and exponential (3 days in RPMI medium containing 10% FCS) cultures. Upper band, cyclin D1 (514 bp); lower band, β-actin (600 bp). Lane 1, DNA molecular weight markers (bp); lane 2, no cDNA (negative control); lanes 3, 5 and 7, OC 314, OC 315 and OC 316 (serum starved); lanes 4, 6 and 8, OC 314, OC 315 and OC 316 (exponential); lane 9, MCF-7 breast cancer cell line

**Figure 6** Cyclin D1 protein levels in ovarian cancer cell lines by Western blotting evaluated in serum-starved (3 days in RPMI medium containing 1% FCS) and exponential (3 days in RPMI medium containing 10% FCS) cultures. Lanes 1, 3 and 5, OC 314, OC 315 and OC 316 (serum starved); lanes 2, 4 and 6, OC 314, OC 315 and OC 316 (exponential); lane 7, MCF-7 breast cancer cell line. The position of the cyclin D1 protein (36 kDa) is reported on the left

**Table 2** Cyclin D1 expression and cell kinetic analysis in ovarian cancer cell lines

| Cell line | Serum starved[a] | | | Exponential[b] | | |
|---|---|---|---|---|---|---|
| | TLI (%) | mRNA[c] | Protein | TLI (%) | mRNA[c] | Protein |
| OC 314 | 8.9 | 0.457 | 0.314 | 55.0 | 0.476 | 0.599 |
| OC 315 | 9.8 | 0.470 | 0.590 | 60.9 | 0.423 | 0.654 |
| OC 316 | 11.6 | 0.487 | 0.568 | 62.8 | 0.480 | 0.299 |

[a]For serum-starved cultures, the cells were grown to subconfluence in RPMI medium containing 10% FCS, the medium was replaced with RPMI 1% FCS for 3 days. [b]For exponential cultures, the cells were plated at $2 \times 10^5$ in 35-mm Petri dishes in RPMI medium containing 10% FCS and grown for 3 days. [c]Cyclin D1 mRNA was normalized to β-actin mRNA values. The MCF-7 cyclin D1-overexpressing cell line densitometric values, used as reference, were 0.598 and 0.400 for mRNA and protein respectively.

 © Cancer Research Campaign 1997

### DISCUSSION

Various genetic alterations have been described in human ovarian cancer, such as amplification of the oncogenes HER-2/*neu*, K-*ras* and c-*myc*, as well as the mutation and overexpression of the p53 tumour-suppressor gene (Piver et al, 1991). Furthermore, chromosome aberrations, including translocations (Lee et al, 1990), rearrangements (Pejovic et al, 1992) and aneuploidy (Rodenburg et al, 1988), have also been reported. As a consequence, concurrent genomic abnormalities could result in alterations of the mechanisms controlling cellular replication at the molecular level, leading to tumour promotion and progression. Although amplification and overexpression of the cyclin D1 gene have been demonstrated in various carcinomas (breast, urinary bladder, head and neck, lung and oesophageal) and play a role in multistep carcinogenesis, data concerning cyclin D1 expression in ovarian cancer are presently lacking.

In the current paper, we reported that the malignant lesions studied differed markedly from the benign lesions in their growth behaviour; moreover, the proliferative activity in ovarian tumours was positively related to the levels of cyclin D1 protein, with D1 being undetectable in benign lesions and highly expressed in carcinomas. Previous studies on cell proliferative activity and DNA flow cytometry of ovarian cancer have demonstrated that highly proliferating and aneuploid cancers are associated with tumour aggressiveness and poor prognosis (Rodenburg et al, 1988; Alama et al, 1994). Therefore, the concomitant analyses of a cell cycle-related gene, such as cyclin D1, with tumour kinetics might provide additional insight into the biology and clinical aggressiveness of this disease.

The relationship between TLI and cyclin D1 expression in the 33 patients with ovarian tumours in this study demonstrated that higher TLI values were significantly associated with cyclin D1 abundance in neoplastic cells, while lower proliferative activity and undetectable cyclin D1 levels were observed in histologically benign ovarian tissues.

The observation that expression of cyclin D1 is frequently related to increased DNA synthesis is in agreement with data from the literature reporting that $G_1$ cyclins are rate controlling for $G_1$ duration and S progression. Indeed, overexpression of $G_1$ cyclins in human fibroblasts 'in vitro' accelerated $G_1$ progression, shortening $G_1$ phase length and reducing serum requirement for the transition from $G_1$ to S (Pagano et al, 1994). Similar results were reported in a paper by Jiang et al (1993*b*) in which retrovirus-transducted Rat 6 embryo fibroblasts, which stably overexpress the human cyclin D1 cDNA, displayed abnormalities in cell cycle and growth control, together with a decrease in the duration of the $G_1$ phase which resulted in shortening their rates of $G_1$ to S-phase transition.

At the clinical level, some preliminary studies have shown that cyclin D1 could represent a feature of malignancy with prognostic significance. One of these reports showed that aberrant expression of cyclin D1 is involved in human hepatocarcinogenesis and, in a subset of five patients with stage IV hepatocellular carcinomas (HCCs), seemed to be associated with faster tumour growth and aggressive behaviour than early-stage disease, for which no alterations of the cyclin D1 gene were detected (Nishida et al, 1994). Moreover, the prognostic significance of cyclin D1 abnormalities was investigated in squamous cell carcinomas of the head and neck (HNSCC) in a study by Michalides et al (1995). The study showed that cyclin D1 overexpression, detected by immunohistochemistry in a retrospective series of 47 operable HNSCC, correlated with rapid cancer recurrence and a poor survival. In addition, as no correlations between cyclin D1 expression and other clinical features (tumour size, stage) were reported, it was suggested that cyclin D1 could represent an independent prognostic marker.

The pathogenetic relevance of cyclin D1 abundance in breast cancer has also been described in detail. One of the first immunohistochemical studies (Bartkova et al, 1994) analysing the cyclin D1 protein in human samples indicated significant differences in the staining intensities between normal tissues and breast carcinomas; 37% of carcinomas revealed a marked detectable cyclin D1 nuclear signal, while normal breast tissues were mainly negative. In addition, a more recent paper by Bartkova et al (1995), including a larger number of tumours (breast, colorectal, uterine, melanoma and soft tissue sarcoma) and their normal counterparts, showed that cyclin D1 immunostaining in carcinomas ranged from weak to high according to the degree of malignancy. The authors concluded that alterations of cyclin D1 expression represented a common feature of malignancy in different human cancers.

The present paper suggests that increased expression of cyclin D1 and high percentage of S-phase cells might be related to the degree of malignancy in ovarian cancer. However, such observations can not exclude that proliferative abnormalities might influence cyclin D1 expression in ovarian neoplastic cells. Although further investigations are needed to precisely understand the role of cyclin D1 in the mechanisms of tumorigenesis, the present study could provide information on cyclin D1 expression as a proliferation marker in benign and borderline ovarian lesions, thus helping identify subsets of patients at increased risk of developing ovarian carcinoma.

### ACKNOWLEDGEMENTS

The skilful technical assistance of Cristina Bruzzo for the experiments with cell cultures is greatly acknowledged. This work has been supported by grants from Consiglio Nazionale Ricerche (PF, ACRO) and Associazione Italiana per la Ricerca sul Cancro (AIRC).

### REFERENCES

Alama A, Chiara S, Merlo F, Ragni N, Conte PF, Meazza R, Reggiardo G, Ferrari I and Rosso R (1994) Tumour kinetics, response to chemotherapy and survival in primary ovarian cancer. *Eur J Cancer* **30A**: 449–552

Alama A, Barbieri F, Favre A, Cagnoli M, Noviello E, Pedull A'F, Viale M and Ragni N (1996) Establishment and characterization of three new cell lines derived from the ascites of human ovarian carcinomas. *Gynecol Oncol* **62**: 82–88

Baldin V, Lukas J, Marcote MJ, Pagano M and Draetta G (1993) Cyclin D1 is a nuclear protein required for cell cycle progression in G1. *Genes Dev* **7**: 812–821

Bartkova J, Lukas J, Muller H, Lutzhoft D, Strauss M and Bartek J (1994) Cyclin D1 protein expression and function in human breast cancer. *Int J Cancer* **57**: 353–361

Bartkova J, Lukas J, Strauss M and Bartek J (1995) Cyclin D1 oncoprotein aberrantly accumulates in malignancies of diverse histogenesis. *Oncogene* **10**: 775–777

Braunschweiger PG, Poulakos L and Schiffer LM (1976) In vitro labeling and gold activation autoradiography for determination of labeling index and DNA synthesis of solid tumors. *Cancer Res* **36**: 1748–1753

Buckley MF, Sweeney KJE, Hamilton JA, Sini RL, Manning DL, Nicholson RI, de Fazio A, Watts CKW, Musgrove EA and Sutherland RL (1993) Expression and amplification of cyclin genes in human breast cancer. *Oncogene* **8**: 2127–2133

Hinds PW, Dowdy SF, Eaton NE, Arnold A and Weinberg RA (1994) Function of a human cyclin gene as an oncogene. *Proc Natl Acad Sci USA* **91**: 709–713

© Cancer Research Campaign 1997 

Hunter T and Pines J (1994) Cyclins and cancer II: cyclin D and CDK inhibitors come of age. *Cell* **79**: 573–582

Jiang W, Zhang YJ, Kahn SM, Hollstein MC, Santella RM, Lu SH, Harris CC, Montesano R and Weinstein IB (1993*a*) Altered expression of the cyclin D1 and retinoblastoma genes in human esophageal cancer. *Proc Natl Acad Sci USA* **90**: 9026–9030

Jiang W, Kahn SM, Zhou P, Zhang Y, Cacace AM, Infante AS, Doi S, Santella RM and Weinstein IB (1993*b*) Overexpression of cyclin D1 in rat fibroblasts causes abnormalities in growth control, cell cycle progression and gene expression. *Oncogene* **8**: 3447–3457

Lammie G and Peters G (1991) Chromosome 11q13 abnormalities in human cancer. *Cancer Cells* **3**: 413–420

Lee JH, Kavanagh JJ, Widrics DM, Wharton JT and Blick M (1990) Frequent loss of heterozygosity on chromosomes 6q, 11, and 17 in human ovarian carcinomas. *Cancer Res* **50**: 2724–2728

Lovec H, Sewing A, Lucibello FC, Muller R and Moroy T (1994) Oncogenic activity of cyclin D1 revealed through cooperation with Ha-ras: link between cell cycle control and malignant transformation. *Oncogene* **9**: 323–326

Lukas J, Pagano M, Staskovic Z, Draetta G and Bartek J (1994) Cyclin D1 protein oscillates and is essential for cell cycle progression in human tumour cell lines. *Oncogene* **9**: 707–718

Michalides R, Van Veelen N, Hart A, Loftus B, Wientjens E and Balm A (1995) Overexpression of cyclin D1 correlates with recurrence in a group of forty-seven operable squamous cell carcinomas of the head and neck. *Cancer Res* **55**: 975–978

Motokura T and Arnold A (1993) Cyclins in oncogenesis. *Biochim Biophys Acta* **1155**: 63–78

Motokura T, Bloom T, Goo Kim H, Juppner H, Ruderman JV, Kronenberg HM and Arnold A (1991) A novel cyclin encoded by a bcl 1-linked candidate oncogene. *Nature* **350**: 512–515

Nishida N, Fukuda Y, Komeda T, Kita R, Sando T, Furukawa M, Amenomori M, Shibagaki I, Nakao K, Ikenaga M and Ishizaki K (1994) Amplification and overexpression of the cyclin D1 gene in aggressive human hepatocellular carcinoma. *Cancer Res* **54**: 3107–3110

Pagano M, Theodoras AM, Tam SW and Draetta G (1994) Cyclin D1-mediated inhibition of repair and replicative DNA synthesis in human fibroblasts. *Genes Dev* **8**: 1627–1639

Peeper DS, Van Der Eb AJ and Zantema A (1994) The G1/S cell-cycle checkpoint in eukaryotic cells. *Biochim Biophys Acta* **1198**: 215–230

Pejovic T, Heim S, Mandahl N, Baldentorp B, Elmfors B, Floderus UM, Furgyik S, Helm G, Himmelman A, Willen H and Mitelman F (1992) Chromosome aberration in 35 primary ovarian carcinomas. *Genes Chrom Cancer* **4**: 58–68

Perez RP, Godwin AK, Hamilton TC and Ozols RF (1991) Ovarian cancer biology. *Semin Oncol* **9**: 1668–1674

Pines J (1993) Cyclins and cyclin-dependent kinases: take your partners. *Trends Biochem Sci* **18**: 195–197

Piver MS, Baker TR, Piedimonte M and Sandecki AM (1991) Epidemiology and etiology of ovarian cancer. *Semin Oncol* **18**: 177–185

Rodenburg CJ, Cornelisse CJ, Hermans J and Fleuren GJ (1988) DNA flow cytometry and morphometry as prognostic indicators in advanced ovarian cancer: a step forward in predicting the clinical outcome. *Gynecol Oncol* **29**: 176–187

Rosenberg CL, Wong E, Petty EM, Bale AE, Tsujimoto Y, Harris NL and Arnold A (1991) PRAD1, a candidate BCL1 oncogene: mapping and expression in centrocytic lymphoma. *Proc Natl Acad Sci USA* **88**: 9638–9642

Schuuring E, Verhoeven E, Mooi WJ and Michalides RJAM (1992) Identification and cloning of two overexpressed genes, U21B31/PRAD1 and EMS1, within the amplified chromosome 11q13 region in human carcinomas. *Oncogene* **7**: 355–361

Ulfelder H (Chairman) (1978) Staging system for cancer at gynecologic sites. In *Manual for Staging of Cancer*, pp. 94–97. JB Lippincott: Philadelphia

Withers DA, Harvey RC, Faust JB, Melnyk O, Carey K and Meeker TC (1991) Characterization of a candidate bcl-1 gene. *Mol Cell Biol* **11**: 4846–4853

*© Cancer Research Campaign 1997*